# Characteristics of suicidal poisoned patients admitted to tertiary care center during COVID-19 pandemic

**DOI:** 10.1186/s41983-022-00577-4

**Published:** 2022-11-22

**Authors:** Ghada El-Sarnagawy, Amal Hafez, Reham Amer

**Affiliations:** 1 Department of Forensic Medicine and Clinical Toxicology, Faculty of Medicine, Tanta University, Tanta, Egypt; 2 Neuropsychiatry Department, Faculty of Medicine, Tanta University, Medical Campus, Al-Gaish Street, Tanta, 31527 Gharbia Egypt

**Keywords:** COVID-19, Poisoning, Suicide, Consultation–liaison psychiatry

## Abstract

**Background:**

Suicidal poisoning is a major concern during the COVID-19 pandemic that has several physical and mental hazards. This study aimed to evaluate the characteristics of suicidal poisoned patients admitted to a tertiary poison control center during the pandemic lockdown and assess COVID-related knowledge and attitude among those patients to identify the high-risk group for suicide. This cross-sectional study was conducted on acutely poisoned patients admitted to Tanta University Poison Control Center from June to December 2020. Upon admission, socio-demographic data, causative poisoning agents, COVID-related knowledge and attitude, Hamilton Anxiety Rating Scale (HAM-A), and Hamilton Depression Rating Scale (HAM-D) were collected from all participants.

**Results:**

A total of 254 poisoned patients were categorized into suicidal (85.04%) and accidental (14.96%) poisoning groups. The former was caused mainly by phosphides and was significantly associated with a history of using psychotropic medications and high HAM-A and HAM-D results. Logistic regression analysis showed that a history of psychiatric illness, low attitude scores, and high HAM-D scores were significant risk factors for suicidal poisoning.

**Conclusions:**

Considerable number of suicidal poisoned patients had moderate-to-severe depressive symptoms, highlighting the importance of providing specialized psychiatric services in poisoning centers, particularly among vulnerable populations, to prevent the overwhelming repeated suicidal attempts.

**Supplementary Information:**

The online version contains supplementary material available at 10.1186/s41983-022-00577-4.

## Background

By March 2020, the World Health Organization (WHO) affirmed the COVID-19 virus as a global pandemic [1] that has various manifestations ranging from flu-like symptoms to severe shortness of breath and even death [2]. The COVID-19 pandemic may cause unfavorable psychosocial, occupational, and economic consequences on individuals and entire communities, especially in developing countries [3]. After the nationwide lockdown, fear of infection and economic instability increased the psychological impact of the COVID-19 pandemic [4]. Hence, many psychiatric disturbances can emerge, ranging from mild anxiety to severe depression with suicidal attempts [5]**.**

During the pandemic era, a recent study [6] in the United States reported a significant association between stress exposure and suicidal attempts due to social isolation and panic fear from COVID-19 infection. Parallel to these findings, Yard and colleagues [7] reported increased suicidal attempt emergency visits in the United States by 31% during 2020 in the adolescents’ age group (between 12 and 17 years), especially among females, compared with the equivalent period in 2019.

Although hanging, firearms, and falling from height are common suicidal ways, self-poisoning is the most widely spread suicidal manner due to its ease of achievement and low suffering [8]**.** Substantially, drug overdoses represented 20% of suicide methods in the USA, and 70% of suicidal deaths were drug abusers [9]. Due to multiple behavioral changes during the COVID-19 breakdown, the incidence of acute poisoned cases admitted to various poison centers dramatically increased [10]. In France, Le Roux and colleagues [11] demonstrated a change in poisoning pattern with a significant increase in emergency calls in the French Poison Control Center during the COVID-19 lockdown period due to personal isolation and anxiety disorders. Furthermore, a previous study [12] in Tanta University Poison Control Center (TUPCC) documented a significant increase in adverse outcomes and mortality rates during the lockdown period. In addition, a micromort analysis by Lee and colleagues [13] in British Columbia demonstrated a significant increase in mortality due to illicit drug overdose than mortality caused by COVID-19 itself (1 miromorts per day versus 0.5 micromorts per day, respectively); they attributed the increased rate of overdose to COVID-19-induced threatening crisis in economic status, social support, and mental health.

However, the argument of psychological effects that influence the pattern of acute poisoning is still limited and less objective. This study was conducted to (1) evaluate the demographic and clinical characteristics of suicidal poisoned patients admitted to TUPCC during the COVID-19 pandemic lockdown; (2) evaluate the knowledge and attitude of poisoned patients toward the COVID-19 pandemic; and (3) to identify the criteria for stratification of high-risk patients for suicide to prevent repeated suicidal poisoning attempts and decrease economic burden, especially with limited hospital resources in this era.

## Methods

This cross-sectional study was conducted on a selected group of acutely poisoned patients admitted to TUPCC from June to December 2020. Our institute’s authorized Medical Research Ethical Committee has approved the study with the code number (33808/5/20). Privacy and confidentiality of patients’ records and data were maintained using coding numbers for every participant. Written informed consents were obtained from all studied patients after fully explaining the study goals.

Selection criteria included all acutely poisoned patients ≥ 18 years of both sexes admitted to our institute during COVID-19 lockdown throughout the study duration. The patient’s diagnosis was based mainly on history taking, clinical presentation as well as the available routine and toxicological laboratory tests. Meanwhile, COVID-19 patients, cases unwilling to participate, and those who presented with any missed data were excluded from this study.

Socio-demographic data (age, sex, marital state, educational level, occupation, residence, monthly salary, and history of medical disease or psychiatric illness) and type of causative poisoning were recorded upon admission. Patients were categorized according to exposure mode into suicidal and accidental poisoning groups. The accidental group included those with accidental poisoning and overdose by addicts (as addicts did not intend to kill themselves).

Types of causative agents were classified into pharmaceutical drugs and non-pharmaceutical ones. The former included central nervous system (CNS) drugs (sedative-hypnotics, antidepressants, antipsychotics, anticonvulsants, and tramadol), cardiac drugs (beta channel blockers and cardiac glycosides), and a miscellaneous group including oral hypoglycemic, analgesics, and antibiotics. The non-pharmaceuticals consisted of pesticides (cholinesterase inhibitors, phosphides, and herbicides), alcohols, hydrocarbon, and food poisoning.

After collecting the abovementioned data, all participants were subjected to psychiatric evaluation using the Arabic version of the Mini international neuropsychiatric interview [14]**.** Comprehensive psychometric tests were done for all patients to assess depressive symptoms by applying the Arabic validated versions of the Hamilton Depression Rating Scale (HAM-D), which was used to measure the severity of depressive symptoms as determined by mood, somatic symptoms, suicidal thoughts, sleep rhythm, psychomotor activity, weight change, anxiety, and guilt feeling [15, 16]**.** Meanwhile, the severity of anxiety symptoms was estimated by the Arabic version of the Hamilton Anxiety Rating Scale (HAM-A), which assesses both psychic and somatic anxiety symptoms [17, 18].

The data concerning the general knowledge and attitude of poisoned patients toward the COVID-19 pandemic were evaluated using the modified knowledge and attitudes questionnaires [19–21].

The knowledge scoring system consists of 19 questions, including five questions about the cause of COVID-19, its incubation period, and its mode of transmission; five questions about COVID-19 symptomatology; five questions about the preventive measures, and lastly, four questions about its treatment. One point was assigned to the corrected answer in each question, while zero point was given to the incorrect /unknown answers. The score values ranged from a minimum score of "zero" to a maximum of "19". Based on the total knowledge score, patients were grouped into good knowledge (score ≥ 12, total score ≥ 60%) or poor knowledge (score < 12, total score < 60%) groups [22].

Patient’s attitude toward the COVID-19 pandemic was assessed using 18 questions that comprised: perception of disease severity (one question), adherence to safety instructions (one question), using protective measures (eight questions), causes of non-use of protective measures (five questions), and lastly coping with the pandemic (three questions). A total attitude score was calculated and ranged from 0 to 15 points, with higher scores indicating a good attitude toward COVID-19. Patients were categorized as having a good or poor attitude if they had more or less than 80% (13 points) of the attitude score, respectively [22].

### Statistical analysis

The sample size was calculated using the equation recommended by Peduzzi and colleagues [23] for a minimum number of cases to include in a study that will conduct logistic regression analysis: *N* = 10 *k*/*p*, where *p* is the smallest of proportions of negative or positive cases in the population, while *k* is the number of independent variables. We assumed that the regression model might include 7 independent variables. The proportion of positive cases in the population (suicidal poisoning cases in our institution) was 0.448 (44.8%), as derived from a study by Abo El-Noor [24]. Therefore, the minimum sample size was *N* = 10 × 7/0.448 = 156 patients.

The internal consistency of the questionnaire sections of knowledge and attitude were assessed in a pilot study on 20 participants (who were not included in the final analysis of the main study). The calculated Cronbach’s alpha was 0.730, which indicates an acceptable level of internal consistency.

The collected data were analyzed using the Statistical Package for Social Sciences for Windows (IBM Corp. Released 2019. IBM SPSS Statistics for Windows, Version 26.0. Armonk, NY: IBM Corp).

The Shapiro–Wilk test for normality was used to assess the distribution of continuous numerical variables. The age variable followed the normal distribution and was summarized as mean and standard deviation (SD), and comparisons between the accidental and suicidal groups were performed using the independent samples T-test. Variables not following the normal distribution (including the knowledge and attitude scores as well as Hamilton’s scales) were summarized as the median and inter-quartile range (IQR, expressed as 25^th^–75th percentiles), and comparisons were performed using the Mann–Whitney test. Correlations between the scores were performed using Spearman’s rank-order correlation. Categorical variables—such as sex (male/female), knowledge (good/poor), and attitude (good/poor)—were summarized as frequencies. Pearson’s Chi-square test for independence, Fisher’s exact test, or Fisher–Freeman–Halton exact test were used to assess the association between the mode of poisoning and categorical variables. A *p* value < 0.05 was adopted to interpret the significance of the statistical tests. A backward elimination binomial logistic regression analysis was performed to assess factors significantly contributing to suicidal poisoning. The variables were entered into the initial model depending on clinical relevance and a *p* value in the univariate analysis below 0.1.

## Results

Two hundred fifty-four eligible adult patients with acute poisoning were admitted to our Poison Control Center during the study period. Of all patients, 216 (85.04%) were caused by suicidal poisoning, while the other 38 (14.96%) were accidental exposures. The mean age of the patients was 25.9 ± 10 years (ranging from 18 to 63 years), and 55.1% were females, with 72.4% from rural areas. Most patients (61%) were single and had secondary-school education (61.8%). Past medical history was positive for: organic diseases in 26%, psychiatric illness in 17.3%, substance abuse in 15%, and psychotropic medication usage in 14.2% of patients. Compared to accidental exposures, suicidal poisoning was significantly associated with a history of psychiatric diseases (19.4% versus 5.3%; *p* = 0.033) and therapeutic administration of psychiatric drugs (16.2% versus 2.6%; *p* = 0.027), as summarized in Table1.

**Table 1:** Basic patients’ demographics (*n* = 254)

	Total (*n* = 254)		Accidental (*n* = 38)		Suicidal (*n* = 216)		Test statistic	*p*
*Age (years)*								
Mean ± SD (Range)	25.9 ± 10.0 (18.0–63.0)		26.2 ± 9.6 (18.0–47.0)		25.8 ± 10.1 (18.0–63.0)		0.237^a^	0.813
*Sex*								
Male	114	44.9%	16	42.1%	98	45.4%	0.139^b^	0.709
Female	140	55.1%	22	57.9%	118	54.6%		
*Marital status*								
Single	155	61.0%	23	60.5%	132	61.1%	4.975^c^	0.168
Married	93	36.6%	13	34.2%	80	37.0%		
Divorced	3	1.2%	2	5.3%	1	0.5%		
Widow	3	1.2%	0	0.0%	3	1.4%		
*Educational level*								
Illiterate	13	5.1%	3	7.9%	10	4.6%	3.495^c^	0.592
Primary	6	2.4%	1	2.6%	5	2.3%		
Preparatory	17	6.7%	4	10.5%	13	6.0%		
Secondary	157	61.8%	21	55.3%	136	63.0%		
University	52	20.5%	7	18.4%	45	20.8%		
Postgraduate	9	3.5%	2	5.3%	7	3.2%		
*Residence*								
Urban	70	27.6%	11	28.9%	59	27.3%	0.043 ^b^	0.835
Rural	184	72.4%	27	71.1%	157	72.7%		
*Occupation*								
Housewife	52	20.5%	6	15.8%	46	21.3%	4.977^c^	0.377
Private work	13	5.1%	3	7.9%	10	4.6%		
Student	127	50.0%	17	44.7%	110	50.9%		
Worker	13	5.1%	1	2.6%	12	5.6%		
Retired	4	1.6%	0	0.0%	4	1.9%		
Employee	45	17.7%	11	28.9%	34	15.7%		
*Monthly salary*								
Below average	66	26.0%	10	26.3%	56	25.9%	4.145^c^	0.118
Average	159	62.6%	20	52.6%	139	64.4%		
Above average	29	11.4%	8	21.1%	21	9.7%		
*Past medical history*								
Organic diseases	66	26.0%	6	15.8%	60	27.8%	2.415^b^	0.120
Psychiatric diseases	44	17.3%	2	5.3%	42	19.4%	4.538^b^	0.033*
Psychiatric drugs	36	14.2%	1	2.6%	35	16.2%	4.893^c^	0.027*
Substance abuse	38	15.0%	4	10.5%	34	15.7%	0.691^b^	0.406
Poison/other drug exposure	45	17.7%	3	7.9%	42	19.4%	2.957^c^	0.086
*Family history of psychiatric diseases*	37	14.6%	5	13.2%	32	14.8%	0.071^b^	0.789

^a^Independent samples *T* test; ^b^Pearson’s Chi-square test; ^c^Fisher–Freeman–Halton exact test; *n*: number; SD: standard deviation;*significant at *p* < 0.05

The most common causative poison was phosphides (19.7%), followed by carbamates/organophosphorus compounds (17.7%), then antipsychotic drugs (9.1%) and tramadol (7.1%). Suicidal poisoning was significantly linked with a higher percentage of phosphides poisoning (22.7% versus 2.6%; *p* = 0.004), while accidental exposure was significantly associated with a higher prevalence of tramadol poisonings (among drug abusers; 15.8% versus 5.6%; *p* = 0.036) and food poisonings (28.9% versus 0%; *p* < 0.001; Additional file 1: Table S1)

Good knowledge (score ≥ 12) was observed in 82.7% of all patients, with no significant difference between the patients of both groups regarding their responses to questions assessing knowledge about COVID-19 (Table 2 and Additional file 1: Table S2). The most frequent sources of knowledge as reported by the patients were social media (34.6%) and Television (24.4%), with no significant differences ( *p* = 0.672) between the two groups (Additional file 1: Table S3).

**Table 2:** Comparison of the score grading between the studied groups (*n* = 254)

	Total (*n* = 254)		Accidental (*n* = 38)		Suicidal (*n* = 216)		Test statistic	*p*
*Knowledge score*								
Good knowledge (≥ 12)	210	82.7%	34	89.5%	176	81.5%	1.441^a^	0.230
Poor knowledge (< 12)	44	17.3%	4	10.5%	40	18.5%		
*Attitude score*								
Good attitude (≥ 13)	22	8.7%	7	18.4%	15	6.9%	FE	0.030*
Poor attitude (< 13)	232	91.3%	31	81.6%	201	93.1%		
*Hamilton Anxiety Scale*								
Mild (0–17)	171	67.3%	33	86.8%	138	63.9%	7.234^b^	0.046*
Mild to moderate (18–24)	63	24.8%	4	10.5%	59	27.3%		
Moderate to severe (25–30)	12	4.7%	1	2.6%	11	5.1%		
Severe (31–56)	8	3.1%	0	0.0%	8	3.7%		
*Hamilton Depression Scale*								
No depression (0–7)	90	35.4%	21	55.3%	69	31.9%	13.392^b^	0.007*
Mild depression (8–13)	82	32.3%	9	23.7%	73	33.8%		
Moderate depression (14–18)	34	13.4%	7	18.4%	27	12.5%		
Severe depression (19–22)	24	9.4%	1	2.6%	23	10.6%		
Very severe depression (≥ 23)	24	9.4%	0	0.0%	24	11.1%		

^a^Pearson’s Chi-square test; ^b^Fisher–Freeman–Halton exact test; *n*: number; *significant at *p* < 0.05

Patients' responses to questions assessing their attitude toward the COVID-19 pandemic are presented in Table 3. The suicidal group was significantly associated with staying at home (*p* = 0.003), and a substantially higher percentage of them always fail to cope with the COVID-19-related difficulties than their counterparts in the accidental group (44.9% versus 5.3%; *p* = 0.001). Table 2 demonstrates that most of the enrolled patients (91.3%) had poor attitude (score < 13), with a significantly higher percentage among the suicidal group compared to the other one (93.1% versus 81.6%; *p* = 0.030). Although we observed a significantly lower median attitude scores in the suicidal group than in the accidental group (7 versus 10; *p* = 0.010). No significant difference was recorded regarding the knowledge score between the two groups (*p* = 0.451, Additional file 1: Table S4).

**Table 3:** Comparison of the attitude toward COVID-19 pandemic between the studied groups (*n* = 254)

	Total (*n* = 254)		Accidental (*n* = 38)		Suicidal (*n* = 216)		Test statistic	*p*
*Perception of disease severity*								
Not dangerous	12	4.7%	2	5.3%	10	4.6%	0.237^a^	0.946
Moderate danger	46	18.1`%	7	18.4%	39	18.1%		
Very dangerous	196	77.2%	29	76.3%	167	77.3%		
*Using protective measures and adhering to MOH instructions*								
Never	19	7.5%	2	5.3%	17	7.9%	0.225^a^	0.927
Sometimes	134	52.8%	20	52.6%	114	52.8%		
Always	101	39.8%	16	42.1%	85	39.4%		
*Used protective methods*								
Healthy diet exercise	87	34.3%	11	28.9%	76	35.2%	0.558^b^	0.455
Stay at home	129	50.8%	11	28.9%	118	54.6%	8.528^b^	0.003*
Avoid crowds	135	53.1%	19	50.0%	116	53.7%	0.178^b^	0.673
Adhere to distancing	103	40.6%	16	42.1%	87	40.3%	0.045^b^	0.832
Washing disinfecting hands	145	57.1%	23	60.5%	122	56.5%	0.216^b^	0.642
Mask	137	53.9%	22	57.9%	115	53.2%	0.282^b^	0.596
Disinfecting surfaces	119	46.9%	15	39.5%	104	48.1%	0.977^b^	0.323
Mix chlorine with other disinfectants	90	35.4%	15	41.7%	75	37.7%	0.204^b^	0.651
*Causes of non-use of protective measures*								
Non-availability	54	21.3%	9	40.9%	45	34.4%	0.355^b^	0.551
Uncomfortable	55	21.7%	8	36.4%	47	35.9%	0.002^b^	0.965
Not convinced	24	9.4%	2	9.1%	22	16.8%	FE	0.530
Forgetting	47	18.5%	7	31.8%	40	30.5%	0.015^b^	0.904
Work stress	44	17.3	4	18.2%	40	30.5%	1.403^b^	0.236
*COVID-19 impacted your personal and familial life*								
No effect	54	21.3%	8	21.1%	46	21.3%	3.339^b^	0.342
Little effect	34	13.4%	3	7.9%	31	14.4%		
Moderate effect	84	33.1%	17	44.7%	67	31.0%		
Large effect	82	32.3%	10	26.3%	72	33.3%		
*You were able to cope with COVID-19-related difficulties*								
No coping	79	31.1%	7	18.4%	72	33.3%	6.508^b^	0.089
Little coping	46	18.1%	7	18.4%	39	18.1%		
Moderate coping	72	28.3%	10	26.3%	62	28.7%		
Good coping	57	22.4%	14	36.8%	43	19.9%		
*You failed to cope with COVID-19-related difficulties and thought of suicide*								
Never	99	39.0%	22	57.9%	28	13.0%	49.725 ^b^	< 0.001*
Sometimes	55	21.7%	10	26.3%	40	18.5		
Often	50	19.7%	4	10.5%	51	23.6%		
Always	50	19.7%	2	5.3%	97	44.9%		

^a^Fisher–Freeman–Halton exact test; ^b^Pearson’s Chi-square test; *n*: number; *MOH*: Ministry of health. *significant at *p* < 0.05

As regards the psychiatric results, Table 2 reveals that approximately two-thirds (67.3%) of all included patients had mild anxiety, and 7.8% only had moderate-to-severe and severe anxiety symptoms. Concerning the depressive symptoms, about one-third of the patients (32.3%) had mild depression, and a comparable number (32.2%) of them suffered from moderate-to-very severe depression. Compared to accidental group, patients with suicidal poisoning had significantly higher percentages of mild depression (33.8% versus 23.7%; *p* = 0.007), severe depression (10.6% versus 2.6%; *p* = 0.007), very severe depression (11.1% versus 0.0%; *p* = 0.007), as well as moderate-to-severe anxiety (5.1% versus 2.6%; *p* = 0.046). Consequently, patients with suicidal poisoning had significantly higher median HAM-A (10 versus 4; *p* = 0.003) and HAM-D (11 versus 7; *p* = 0.005) scores than patients with accidental poisoning (Additional file 1: Table S4).

**Figure. 1:**
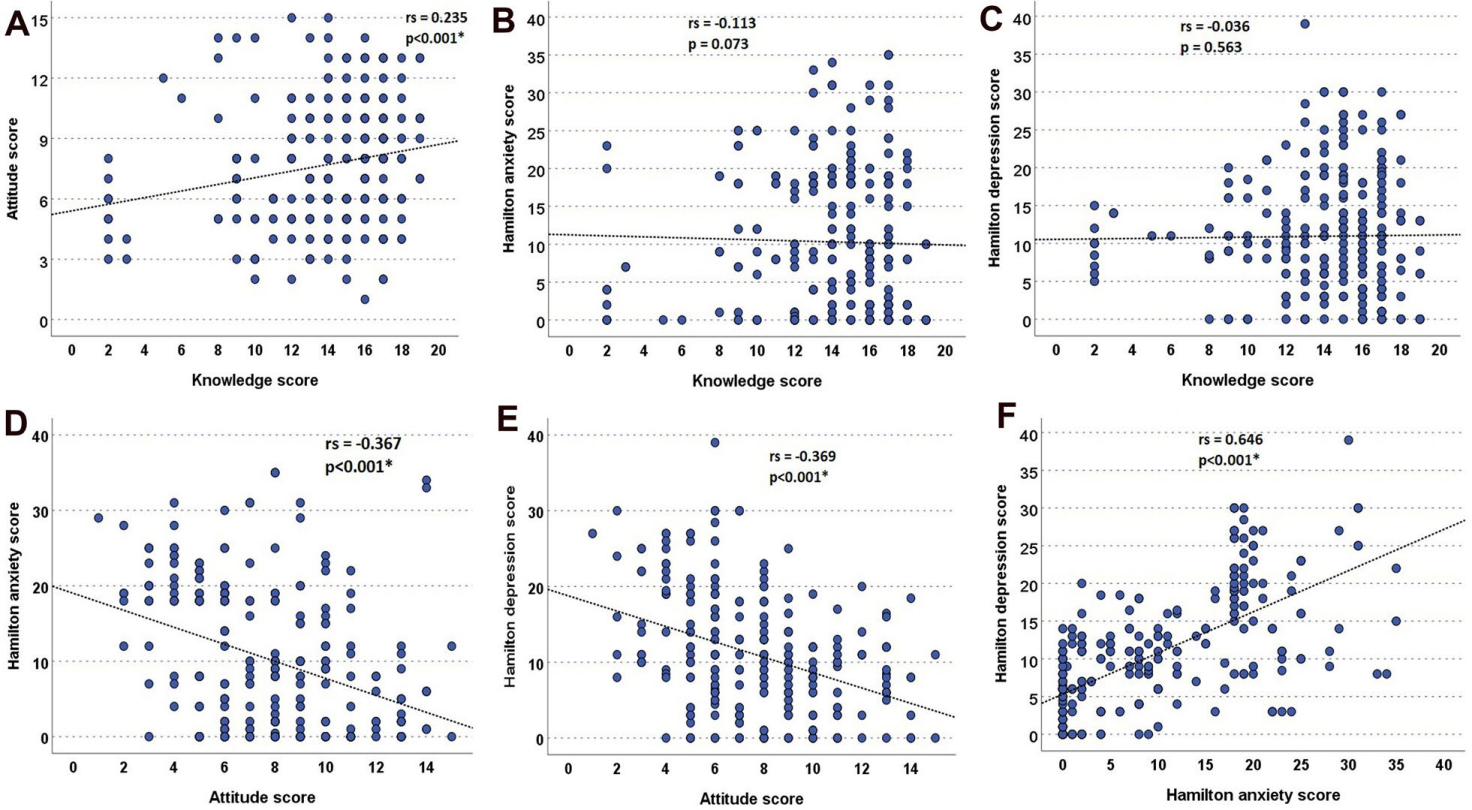
Correlations between the studied scores

The knowledge score had a weak significant positive correlation with the attitude score (rs = 0.235, *p* < 0.001), but did not correlate significantly with HAM-A or HAM-D scores (*p* > 0.05). The attitude scores also showed a significant negative, weak correlation with HAM-A (rs = -0.367, *p* < 0.001) and HAM-D scores (rs = -0.369, *p* < 0.001). The HAM-A and HAM-D scores were correlated significantly, moderately, and positively (rs = 0.646, *p* < 0.001), as illustrated in Fig. 1.

The binomial logistic regression analysis that assessed factors contributing significantly to suicidal poisoning are shown in Table 4. The final model showed that decreased attitude scores (OR: 0.867, 95% CI 0.763–0.986, *p* = 0.030), increased HAM-D scores (OR: 1.059, 95% CI 1.002–1.120, *p* = 0.042), and the presence of a past-history of psychiatric diseases (OR: 5.063, 95% CI 1.151–22.267, *p* = 0.032) were significantly associated with a higher likelihood of suicidal poisoning.

**Table 4:** Backward elimination binomial logistic regression to detect factors impacting suicidal poisoning (*n* = 254)

Variables	OR	95% CI	*p*
*Initial Model*			
Attitude score	0.874	0.767–0.995	0.042*
Hamilton Anxiety Scale	1.028	0.973–1.086	0.325
Hamilton Depression Scale	1.040	0.974–1.111	0.241
Past history of psychiatric diseases	4.869	1.103–21.490	0.037*
*Final Model*			
Attitude score	0.867	0.763–0.986	0.030*
Hamilton Depression Scale	1.059	1.002–1.120	0.042*
Past history of psychiatric diseases	5.063	1.151–22.267	0.032*

*CI* confidence interval, *OR* odds ratio, *Significant at *p* < 0.05

## Discussion

Worldwide, suicide is a major psychological problem among venerable subjects. The WHO reported more than 700,000 suicidal deaths annually, especially in developing countries [25]. The current study demonstrated a high incidence (85.04%) of suicidal poisoning among the included conventional sample admitted during COVID-19 lockdown. In the same line, Shrestha and colleagues [26] reported a significant increase (43.58%) of suicidal poisoning cases in Nepal during the lockdown period compared to an equivalent period in the previous two years (31.03% and 25.64%, respectively). The increased suicidal rates during a pandemic could be attributed to complex mental health troubles that are usually related to the morbidity/mortality of the disease itself, public fear, prolonged social isolation, and economic burden [5, 27]. Conversely, previous studies in Egypt [12] and Italy [28] reported a decrease in the suicidal poisoning incidence during the lockdown period compared to previous years. The authors explained this decline by the decreased number of all admitted poisoning cases throughout the lockdown [12].

A systematic review in Bangladesh [29] highlighted four risk factors for suicidal behavior through the pandemic, including demographic characteristics (female gender, single marital status, and low education levels), behavioral factors (cigarette smoking and substance abuse), COVID-19-related pandemic factors (reduced knowledge and preventive practice levels), and psychiatric susceptibility (high levels of anxiety and depression as well as repeated suicidal attempts). In partial agreement with the abovementioned findings, the present study recorded that suicidal poisoning patients had a significant psychiatric vulnerability, low coping attitude scores, and high scores on both HAM-A and HAM-D scales. Differences in the risk factors across various studies are multifactorial, and could be partially attributed to ethnic and cultural variations among the populations. Mak and colleagues [30] delineated that ethnic difference is influential in both psychiatric illness and unemployment which are well-recognized risk factors for committing suicide. They also emphasized that religiosity, expression of regret, self-motivation to seek help as well as the presence of good social, emotional, and financial supports may play a major protective role in alleviating the risk of suicidal attempts among different races.

Regarding the demographic factors, the results of the current study were concordant with another study in Eastern Nepal [31] which documented that the majority of acutely poisoned patients were within 16–30 years. These results may be explained by the aggravation of various personal and social responsibilities related to this age group during lockdown [32]. In addition, females have a higher prevalence of poisoning as they are more emotionally affected by the consequences of the pandemic [31].

In agreement with our results, Mamun and colleagues [33] found that most acutely poisoned patients were single and more vulnerable to psychological distress and suicidal attempts, especially after the compulsory lockdown. Compared to the accidental group in the current study, the suicidal poisoning group had a significant association with a history of psychiatric illness (19.4% versus 5.3%, *p* = 0.033) and therapeutic psychiatric drug administration (16.2% versus 2.6%, *p* = 0.027). These findings are harmonious with the results of previous researchers [34, 35] who revealed that 13% and 31.6% of suicidal poisoned patients, respectively, had pre-existing psychiatric disorders. Nistor and colleagues [36] also concluded that psychological disturbance increases the risk of suicidal poisoning by 11% to 27% more than the overall population. In the same line, Ho and his colleagues [37] observed that patients who attempted suicide using drug overdose had a higher rate of past psychiatric ward admissions than those who attempted non-overdose suicide. Subsequently, Methling and colleagues [38] recommended monitoring patients with antidepressant and antipsychotic drug therapy for suicidal tendencies to protect them from committing suicide.

Consistent with previous studies [12, 39], phosphide pesticides were the most frequent cause of suicidal poisoning in our sample. It could be explained by their wide availability owing to uncontrolled sales in our agricultural locality, making them the method of choice for committing suicide [40]. Conversely, pharmaceuticals are commonly used for suicidal poisoning in Europe and Iran [41, 42]. This variation could be attributed to country-to-country substance availability. In addition, the subjects’ knowledge about the substance’s lethal effect and the degree of suicidal intention are other risk factors for suicide [43].

The present study recorded a significant association between accidental poisoning with tramadol overdose. In Egypt, the rate of tramadol abuse has been increasing steadily, especially among young adults [44]. During the pandemic, factors such as panic, fear of infection, economic instability, social isolation, and depression can trigger medication or illicit substance abuse [45]. Although other studies [28, 46] found a decrease in the rate of substance abuse due to limited accessibility, they noted a significant association between accidental poisoning and household cleaners and disinfectants due to the misuse of these products during the pandemic lockdown.

The current study also revealed that prolonged stay at home and failure to cope with COVID-19-related difficulties were significantly associated with suicidal poisoning. Likewise, Jassim and his colleagues [47] emphasized that social isolation and prolonged duration of loneliness may potentiate the development of depression and suicidal thoughts. Eisenbeck and colleagues [48] have recently documented that active coping was linked negatively with psychological distress and depression–anxiety–stress scores.

In agreement with previous studies [49, 50] that assessed the degree of knowledge about the COVID-19 pandemic, the present article showed that both groups had good knowledge levels without any significant difference between the two modes of poisoning (*p* > 0.05). This finding could be attributed to getting sufficient information about the COVID-19 pandemic from social media, and the majority of included patients had secondary school education in our sample. On the other hand, the suicidal poisoned group in this study had significantly lower attitude scores (*p* < 0.05) than their accidentally poisoned counterparts. This result could be explained by the negative attitudes of some patients toward protective measures, which may be related to the psychological reluctance and/or the false concept that masks, gloves and other protective measures are ineffective in preventing COVID-19 [51].

Regarding the coping strategies, previous researches [48, 52] have demonstrated that subjects with bad coping attitudes and maladaptive responses toward stressors had higher anxiety and depressive scores than good adaptors. In the same line, our study verified a significant negative correlation between attitude and both the HAM-A (rs = -0.367, *p* < 0.001) and HAM-D scores (rs = -0.369, *p* < 0.001). Substantially during the COVID-19 pandemic, coping among patients with psychiatric illness was very hard due to the possible interaction of multiple stressors resulting in self-harm and suicidal attempts [26].

Moreover, the current study highlighted that more than a third of suicidal poisoned patients (32.2%) had moderate-to-severe degrees of depression. This result was in concurrence with Dedic and colleagues [53], who recorded that the HAM-D scores were moderate-to-severe in suicidal poisoning subjects, while mild in controls. Furthermore, Nitescu and colleagues [34] noticed that depression represented 36.1% of common psychiatric disorders among adolescents with suicidal poisoning. Similarly, a previous research in China [54] concluded that more than half of their included subjects had moderate-to-severe degrees of both anxiety and depression during the COVID-19 pandemic. Likewise, El-Farouny and Helmy [55] reported that most suicidal poisoned cases had neuroticism personalities, characterized by greater liability for anxiety and depression.

The abovementioned results pointed to the value of evaluating patients’ mental health status by qualified psychiatrists when facing suicidal poisoning to provide the optimal psychiatric services (outpatient versus inpatient services) and to determine pharmacological and non-pharmacological treatment indications. In addition, evaluating patients’ psychological conflicts that lead to suicidal attempts is considered a preliminary basis for the psychiatrist to choose a suitable crisis intervention plan [53]. Well-recognized treatment guidelines for depressive disorders have recommend the use of psychotherapy as monotherapy for mild depressive symptoms and pharmacotherapy (with or without psychotherapy) for moderate and severe depression [56].

It is crucial to identify the criteria of vulnerable patients for suicide who need early psychotherapeutic interventions to improve patients’ outcomes in one hand, and prevent repeated suicidal attempts without wasting financial and human resources on the other hand. Regression analysis results of our study revealed that patients who had a positive history of psychiatric illness, decreased attitude scores, and increased HAM-D scores were significantly associated with a higher likelihood of suicidal poisoning. Similarly, Dedic and colleagues [53] demonstrated that the HAM-D score was the major risk factor of repeated suicide in acutely poisoned patients by logistic regression analysis with (OR: 0.22; 95% CI 0.01–9.48; *p* < 0.001). Correspondingly, the binary logistic regression in an earlier study from Iran [57] showed a significant association between suicidal attempts and the existence of previous psychiatric consultation (OR = 4.290; 95% CI 1.19–15.41; *p* = 0.03).

McIntyre and colleagues [58], based on their exploratory analysis of the Canadian suicide rates during the first year of the pandemic, have suggested an integrated preventive strategies to reduce the suicidal rates during and after the COVID-19 era. This includes provisioning of timely psychiatric services as well as government initiatives (financial, residential, healthcare, public education and outreach programs) to minimize stressors and address all aspects of insecurity. As well, a previous study [37] emphasized the importance of drug legalization with imposing strict barriers to lethal drugs as well as firm control over quantity of widespread medications, that can be bought over the counter, as a national strategy for suicide reduction.

Although psychiatric screening of poisoned patients is well-tolerated, this does not occur in several poison centers. Many toxicologists choose to treat acutely poisoned patients exclusively without seeking assessment (particularly if psychiatric consultation cannot be easily obtained), while others prefer to involve accessible psychiatric services [59, 60]. Lack of management strategies of co-morbid psychiatric symptoms may result in inappropriate or suboptimal treatment of poisoned patients with consequent poor outcomes. Therefore, “consultation–liaison psychiatry,” which involves routine psychiatric screening and treatment, together with the protocol of acute poisoning investigations and management strategies, is strongly recommended in poison control centers.

It is worth mentioning that our study has certain limitations. The absence of a control group of healthy participants, an issue which was hard to achieve during the lockdown, is one of these limitations. In addition, the inequality of group distribution is another shortcoming, which could be attributed to the lower number of admitted accidentally poisoned cases and decreased subject participation among this group during the pandemic. Moreover, the cross-sectional study design, which did not address follow-up psychiatric data and subsequent suicidal attempts is another limitation. Likewise, the study neither included past psychiatric admissions in the statistical analysis, nor explored the role of religion and other protective factors on the results. It is worth noting that in our country, with a predominantly religious society, suicide is considered a sin, and frequent psychiatric admissions could be regarded as mental stigma, which may make it subject to denial and may interfere with precise documentation of this issue. The study depended mainly on the patients’ honesty and recalls, which may be affected by subjective bias.

Several multi-center randomized longitudinal studies on a wider scale of population are strongly recommended to evaluate the definite criteria for psychiatric consultation and treatment to avoid repeated suicidal attempts among suicidal poisoned patients.

## Conclusions

Throughout the COVID-19 pandemic lockdown, our study recorded a high incidence of suicidal poisoned patients; most of them were poisoned with phosphides and had a history of psychiatric disease and the use of psychotropic medications. Contributing factors such as history of psychiatric illness, low coping levels, and high HAM-D scores were associated with a higher likelihood of suicidal poisoning. Therefore, identifying these predictive factors may stratify the high-risk patients for early psychiatric consultation to improve patient's outcomes and prevent repeated suicidal attempts. The ultimate goal should not be limited to treating the poisoned patients during the acute stage, but extend beyond rescue to prevent the risks of such recurring behavior. Therefore, psychiatric staff members should be part of a multidisciplinary team in poison control centers to provide optimal physical and mental health care.

## Supplementary Information

**Additional file 1: Table S1.** Distribution of the toxic substances in the studied groups. **Table S2.** Comparison of the knowledge score between the studied groups. **Table S3.** Sources of knowledge in the studied groups. **Table S4.** Comparison of the studied scores between groups.

## Data Availability

The data that support the findings of this study are available from the corresponding author, [Reham Amer], upon reasonable request.

## Abbreviations

COVID-19: Coronavirus disease 2019
HAM-A: Hamilton Anxiety Rating Scale
HAM-D: Hamilton Depression Rating Scale
TUPCC: Tanta University Poison Control Center
WHO: World Health Organization
