# Supplementary material for: Characteristics of suicidal poisoned patients admitted to tertiary care center during COVID-19 pandemic
Source: Egypt J Neurol Psychiatr Neurosurg. 2022 Nov 22;58(1):138. doi: 10.1186/s41983-022-00577-4 (PMC9684898; doi:10.1186/s41983-022-00577-4)
Supplement: Supplementary file 1 — Additional file 1: Table S1. Distribution of the toxic substances in the studied groups. Table S2. Comparison of the knowledge score between the studied groups. Table S3. Sources of knowledge in the studied groups. Table S4. Comparison of the studied scores between groups. [file 41983_2022_577_MOESM1_ESM.docx]

**Supplementary table 1:** Distribution of the toxic substances in the studied groups (*n* = 254)

|  | **Total**  **(*n* = 254)** | | | **Accidental**  **(*n* = 38)** | | **Suicidal**  **(*n* = 216)** | | **Test statistic** | ***p*** |
| --- | --- | --- | --- | --- | --- | --- | --- | --- | --- |
|  |  | |  |  |  |  |  |  |  |
| **I) Pharmaceutical drugs:** |  | |  |  |  |  |  |  |  |
| *A- CNS drugs* |  | |  |  |  |  |  |  |  |
| - Antipsychotics | 23 | | 9.1% | 7 | 18.4% | 16 | 7.4% | FE | 0.058 |
| - Antidepressants | 2 | | 0.8% | 0 | 0.0% | 2 | 0.9% | FE | 1.000 |
| - Anticonvulsants | 13 | | 5.1% | 3 | 7.9% | 10 | 4.6% | FE | 0.420 |
| - Sedative-hypnotics | 5 | | 2.0% | 2 | 5.3% | 3 | 1.4% | FE | 0.163 |
| - Tramadol | 18 | | 7.1% | 6 | 15.8% | 12 | 5.6% | FE | 0.036* |
| *B- CVS drugs* |  | |  |  |  |  |  |  |  |
| - Cardiac glycosides | 8 | | 3.1% | 0 | 0.0% | 8 | 3.7% | FE | 0.610 |
| - Beta blockers | 3 | | 1.2% | 1 | 2.6% | 2 | 0.9% | FE | 0.386 |
| *C- Miscellaneous* |  | |  |  |  |  |  |  |  |
| - Analgesics | 8 | | 3.1% | 0 | 0.0% | 8 | 3.7% | FE | 0.610 |
| - Antibiotics | 2 | | 0.8% | 0 | 0.0% | 2 | 0.9% | FE | 1.000 |
| - Anticoagulants | 4 | | 1.6% | 2 | 5.3% | 2 | 0.9% | FE | 0.108 |
| - Oral hypoglycemic | 12 | | 4.7% | 0 | 0.0% | 12 | 5.6% | FE | 0.223 |
| - Iron | 16 | | 6.3% | 0 | 0.0% | 16 | 7.4% | FE | 0.140 |
| - Theophylline | 14 | | 5.5% | 0 | 0.0% | 14 | 6.5% | FE | 0.138 |
| **II) Non-pharmaceuticals:** |  | |  |  |  |  |  |  |  |
| - Household disinfectants | | 16 | 6.3% | 3 | 7.9% | 13 | 6.0% | FE | 0.715 |
| - Herbicides | | 10 | 3.9% | 1 | 2.6% | 9 | 4.2% | FE | 1.000 |
| - Phosphides | | 50 | 19.7% | 1 | 2.6% | 49 | 22.7% | 8.220 ^a^ | 0.004* |
| - Carbamates/OP | | 45 | 17.7% | 4 | 10.5% | 41 | 19.0% | 1.585 ^a^ | 0.208 |
| - Food poisonings | | 11 | 4.3% | 11 | 28.9% | 0 | 0.0% | FE | <0.001* |

a:Pearson’s Chi-square test; FE:Fisher’s exact test; n: number;*significant at *p*<0.05

**Supplementary table 2:** Comparison of knowledge score between the studied groups (*n* = 254)

|  | | **Total**  **(*n* = 254)** | | | **Accidental**  **(*n* = 38)** | | | **Suicidal**  **(*n* = 216)** | | | **Test statistic** | | ***p*** | |  |
| --- | --- | --- | --- | --- | --- | --- | --- | --- | --- | --- | --- | --- | --- | --- | --- |
| Cause of disease | Incorrect | | 58 | 22.8% | | 9 | 23.7% | | 49 | 22.7% | | 0.018 ^a^ | | 0.892 | |
|  | Correct | | 196 | 77.2% | | 29 | 76.3% | | 167 | 77.3% | |  |  |  |  |
| Disease transmission | Incorrect | | 46 | 18.1% | | 7 | 18.4% | | 39 | 18.1% | | 0.003 ^a^ | | 0.957 | |
|  | Correct | | 208 | 81.9% | | 31 | 81.6% | | 177 | 81.9% | |  |  |  |  |
| Incubation period | Incorrect | | 82 | 32.3% | | 15 | 39.5% | | 67 | 31.0% | | 1.057 ^a^ | | 0.304 | |
|  | Correct | | 172 | 67.7% | | 23 | 60.5% | | 149 | 69.0% | |  |  |  |  |
| The disease transfers via pets | Incorrect | | 93 | 36.6% | | 15 | 39.5% | | 78 | 36.1% | | 0.157 ^a^ | | 0.692 | |
|  | Correct | | 161 | 63.4% | | 23 | 60.5% | | 138 | 63.9% | |  |  |  |  |
| The disease transfers via infected individuals | Incorrect | | 37 | 14.6% | | 6 | 15.8% | | 31 | 14.4% | | 0.054 ^a^ | | 0.817 | |
|  | Correct | | 217 | 85.4% | | 32 | 84.2% | | 185 | 85.6% | |  |  |  |  |
| Covid-19 has upper respiratory symptoms | Incorrect | | 46 | 18.1% | | 4 | 10.5% | | 42 | 19.4% | | 1.733 ^a^ | | 0.188 | |
|  | Correct | | 208 | 81.9% | | 34 | 89.5% | | 174 | 80.6% | |  |  |  |  |
| Covid-19 has lower respiratory symptoms | Incorrect | | 97 | 38.2% | | 16 | 42.1% | | 81 | 37.5% | | 0.290 ^a^ | | 0.590 | |
|  | Correct | | 157 | 61.8% | | 22 | 57.9% | | 135 | 62.5% | |  |  |  |  |
| Covid-19 causes fever and muscle pain | Incorrect | | 52 | 20.5% | | 9 | 23.7% | | 43 | 19.9% | | 0.283 ^a^ | | 0.595 | |
|  | Correct | | 202 | 79.5% | | 29 | 76.3% | | 173 | 80.1% | |  |  |  |  |
| Covid-19 has digestive symptoms | Incorrect | | 120 | 47.2% | | 16 | 42.1% | | 104 | 48.1% | | 0.473 ^a^ | | 0.491 | |
|  | Correct | | 134 | 52.8% | | 22 | 57.9% | | 112 | 51.9% | |  |  |  |  |
| Covid-19 has CNS symptoms | Incorrect | | 109 | 42.9% | | 16 | 42.1% | | 93 | 43.1% | | 0.012 ^a^ | | 0.913 | |
|  | Correct | | 145 | 57.1% | | 22 | 57.9% | | 123 | 56.9% | |  |  |  |  |
| Washing hands with alcohol can prevent disease | Incorrect | | 21 | 8.3% | | 2 | 5.3% | | 19 | 8.8% | | FE | | 0.749 | |
|  | Correct | | 233 | 91.7% | | 36 | 94.7% | | 197 | 91.2% | |  |  |  |  |
| Covering nose & mouth can prevent disease | Incorrect | | 26 | 10.2% | | 2 | 5.3% | | 24 | 11.1% | | FE | | 0.389 | |
|  | Correct | | 228 | 89.8% | | 36 | 94.7% | | 192 | 88.9% | |  |  |  |  |
| Maintaining good general health can prevent disease | Incorrect | | 32 | 12.6% | | 3 | 7.9% | | 29 | 13.4% | | FE | | 0.436 | |
|  | Correct | | 222 | 87.4% | | 35 | 92.1% | | 187 | 86.6% | |  |  |  |  |
| Vaccination can prevent disease | Incorrect | | 105 | 41.3% | | 15 | 39.5% | | 90 | 41.7% | | 0.064 ^a^ | | 0.800 | |
|  | Correct | | 149 | 58.7% | | 23 | 60.5% | | 126 | 58.3% | |  |  |  |  |
| There are no preventive measures | Incorrect | | 106 | 41.7% | | 19 | 50.0% | | 87 | 40.3% | | 1.256 ^a^ | | 0.262 | |
|  | Correct | | 148 | 58.3% | | 19 | 50.0% | | 129 | 59.7% | |  |  |  |  |
| Supportive treatment at home is a line of treatment | Incorrect | | 58 | 22.8% | | 6 | 15.8% | | 52 | 24.1% | | 1.259 ^a^ | | 0.262 | |
|  | Correct | | 196 | 77.2% | | 32 | 84.2% | | 164 | 75.9% | |  |  |  |  |
| Hospital admission is a line of treatment | Incorrect | | 48 | 18.9% | | 7 | 18.4% | | 41 | 19.0% | | 0.007 ^a^ | | 0.935 | |
|  | Correct | | 206 | 81.1% | | 31 | 81.6% | | 175 | 81.0% | |  |  |  |  |
| ICU admission is a line of treatment | Incorrect | | 45 | 17.7% | | 4 | 10.5% | | 41 | 19.0% | | 1.585 ^a^ | | 0.208 | |
|  | Correct | | 209 | 82.3% | | 34 | 89.5% | | 175 | 81.0% | |  |  |  |  |
| There is no available specific drug therapy | Incorrect | | 83 | 32.7% | | 8 | 21.1% | | 75 | 34.7% | | 2.745 ^a^ | | 0.098 | |
|  | Correct | | 171 | 67.3% | | 30 | 78.9% | | 141 | 65.3% | |  |  |  |  |

a: Pearson’s Chi-square test; FE: Fisher’s exact test; n: number; *significant at p<0.05

**Supplementary table 3:** Sources of knowledge in the studied group (*n* = 254)

|  | **Total**  **(*n* = 254)** | | **Accidental**  **(*n* = 38)** | | **Suicidal**  **(*n* = 216)** | | **Fisher-Frteeman-Halton exact test** | ***p*** |
| --- | --- | --- | --- | --- | --- | --- | --- | --- |
|  |  |  |  |  |  |  |  |  |
| **Physicians** | 19 | 7.5% | 5 | 13.2% | 14 | 6.5% | 3.847 | 0.672 |
| **Social media** | 88 | 34.6% | 11 | 28.9% | 77 | 35.6% |  |  |
| **Newspapers** | 2 | 0.8% | 0 | 0.0% | 2 | 0.9% |  |  |
| **TV** | 62 | 24.4% | 10 | 26.3% | 52 | 24.1% |  |  |
| **Educational campaigns** | 8 | 3.1% | 1 | 2.6% | 7 | 3.2% |  |  |
| **Do not know** | 8 | 3.1% | 2 | 5.3% | 6 | 2.8% |  |  |
| **Multiple sources** | 67 | 26.4% | 9 | 23.7% | 58 | 26.9% |  |  |

**Supplementary table 4:** Comparison of the studied scores between groups (*n* = 254)

|  | | **Total**  **(*n* = 254)** | **Accidental**  **(*n* = 38)** | **Suicidal**  **(*n* = 216)** | **Mann-Whitney test** | ***p*** |
| --- | --- | --- | --- | --- | --- | --- |
|  |  |  |  |  |  |  |
| **Knowledge score** | Median [IQR]  (range) | 15 [13 - 17]  (2 - 19) | 16 [13 - 17]  (3 - 18) | 15 [13 - 17]  (2 - 19) | 0.754 | 0.451 |
|  | Mean rank |  | 135.8 | 126.1 |  |  |
|  |  |  |  |  |  |  |
| **Attitude score** | Median [IQR]  (range) | 8 [6 - 10]  (1 - 15) | 10 [5 - 11]  (2 - 15) | 7 [6 - 9]  (1 - 14) | 2.574 | 0.010* |
|  | Mean rank |  | 155.6 | 122.6 |  |  |
|  |  |  |  |  |  |  |
| **Hamilton Anxiety Scale** | Median [IQR]  (range) | 8 [1 - 18]  (0 - 35) | 4 [0 - 8]  (0 - 28) | 10 [2 - 19]  (0 - 35) | 3.021 | 0.003* |
|  | Mean rank |  | 94.5 | 133.3 |  |  |
|  |  |  |  |  |  |  |
| **Hamilton Depression Scale** | Median [IQR]  (range) | 10 [5 - 16]  (0 - 39) | 7 [3 - 11]  (0 - 20) | 11 [6 - 17]  (0 - 39) | 2.776 | 0.005* |
|  | Mean rank |  | 97.0 | 132.9 |  |  |

IQR: interquartile range; n: number; *significant at *p*<0.05
